# The Role of Galectin-3 Levels for Predicting Paroxysmal Atrial Fibrillation in Patients with Embolic Stroke of Undetermined Source

**DOI:** 10.3390/jcm13113175

**Published:** 2024-05-29

**Authors:** Bekir Çalapkorur, Erkan Demirci, Oğuzhan Baran, Ersin Kasım Ulusoy, Derya Koçer, Selami Demirelli, Mustafa Gök, Ziya Şimşek

**Affiliations:** 1 Department of Cardiology, Kayseri City Education and Research Hospital, Kayseri 38080, Turkey; demirci.e@hotmail.com (E.D.); oguzhanbaran2009@hotmail.com (O.B.); demirelli23@yahoo.com (S.D.); mustafagok2010@gmail.com (M.G.); ziyamposta@hotmail.com (Z.Ş.); 2 Department of Neurology, Ankara City Education and Research Hospital, Ankara 38080, Turkey; ersinkasim_ulusoy@hotmail.com; 3 Department of Biochemistry, Kayseri City Education and Research Hospital, Kayseri 38080, Turkey; ayder78@yahoo.com

**Keywords:** embolic stroke of undetermined source, galectin-3, left atrial strain, paroxysmal atrial fibrillation, holter electrocardiography monitoring

## Abstract

**Background/Objectives:** Paroxysmal atrial fibrillation (PAF) is an important cause that is thought main potential factor in Embolic stroke of undetermined source (ESUS). Extended Holter ECG is an expensive and time-consuming examination. It needs another tools for predicting PAF in ESUS patients. In this study, serum galectin-3 levels, ECG parameters (PR interval, P wave time and P wave peak time) LA volume index, LA global peak strain and atrial electromechanical conduction time values were investigated for predicting PAF. **Methods:** 150 patients with ESUS and 30 volunteers for the control group were recruited to study. 48–72 h Holter ECG monitoring was used for detecting PAF. Patients were divided into two groups (ESUS + PAF and ESUS-PAF) according to the development of PAF in Holter ECG monitoring. **Results:** 30 patients with ESUS whose Holter ECG monitoring showed PAF, were recruited to the ESUS + PAF group. Other 120 patients with ESUS were recruited to the ESUS-PAF group. PA lateral, PA septum, and PA tricuspid were higher in the ESUS + PAF group (*p* < 0.001 for all). Serum galectin-3 levels were significantly higher in ESUS + PAF than in ESUS-PAF and control groups (479.0 pg/mL ± 435.8 pg/mL, 297.8 pg/mL ± 280.3 pg/mL, and 125.4 ± 87.0 pg/mL, *p* < 0.001, respectively). Serum galectin-3 levels were significantly correlated with LAVI, PA lateral, and global peak LA strain (r = 0.246, *p* = 0.001, *p* = 0.158, *p* = 0.035, r = −0.176, *p* = 0.018, respectively). **Conclusion:** Serum galectin-3 levels is found higher in ESUS patients which developed PAF and Serum galectin-3 levels are associated LA adverse remodeling in patients with ESUS.

## 1. Introduction

Embolic stroke of undetermined source (ESUS) is used for cryptogenic strokes probably due to distal embolization [1]. Because, metanalysis showed the annual recurrence rate of ESUS is 4.5%, detailed evolution and secondary prevention measures for embolic cause are crucial [2]. Paroxysmal atrial fibrillation (PAF) is an important cause that is thought main potential factor in ESUS [3]. However, in ESUS, prolonged cardiac monitoring is recommended for adequately detecting PAF, studies showed that few ESUS patients receive adequate cardiac monitoring [4]. Prolonged cardiac monitoring needs technical and human capability and suitable time. Also, some patients with PAF cannot be diagnosed in prolonged cardiac monitoring, unless AF occurs in the monitoring time [5]. Different easy methods are needed to detect PAF more accurately.

Galectin-3 is a protein that plays a role in the link between cell-to-cell and cell-to-extracellular matrix [6]. Many studies show that galectin-3 is associated with fibrotic processes and cardiac fibrosis [7]. Previously, galectin-3 levels correlated with atrial fibrosis and atrial electromechanical delay (EMD) in PAF [8]. Galectin-3 can be a useful marker for predicting paroxysmal AF.

Left atrium (LA) enlargement facilitates to occurrence of AF. Left atrial volume index (LAVI) is a validating parameter for measuring LA enlargement [9]. It has been shown that LAVI was a useful parameter for AF detection in patients with ESUS [9]. Global peak LA strain and atrial EMD are beneficial parameters for predicting AF in patients with ESUS [10,11].

In electrocardiographic (ECG), the P wave gives information about LA function and morphology. P wave peak time is a novel marker that is associated with PAF in patients with acute ischemic stroke [12]. However, there is limited about whether P wave has an association with PAF in patients with ESUS

In this study, we determined patients with ESUS who developed and did not have PAF by using 48–72 h of Holter ECG monitoring. We examined serum galectin-3 levels, ECG parameter (PR interval, P wave time and P wave peak time) and echocardiographic parameters (LA volume index, LA global peak strain and atrial electromechanical conduction time values) for investigating to association between these parameters and PAF. The role of these parameters for predicting PAF in ESUS patients were investigated. The association between serum galectin-3 levels and ECG and echocardiographic parameters was determined. Also, these parameters were compared in ESUS patients and the control group.

## 2. Materials and Methods

The study is a prospective cohort study involving diagnosed ESUS patients who applied to the neurology and cardiology department. The patients’ baseline demographics, clinical ECG, and transthoracic echocardiography characteristics were recorded. All patients underwent a detailed physical examination. Questions were asked to all patients to record their age, gender, diabetes, hyperlipidemia, hypertension, heart failure, history of coronary artery disease, and other accompanying diseases. The study was approved by the local ethics committee (approval No: 395). The written consents were obtained from all patients. The study complied with the ethical principles of the Helsinki Declaration.

150 patients (69 female 46%, age: 64.4 ± 11.2) who were hospitalized and diagnosed with stroke and ESUS, were recruited to study. Age and sex-matched 30 volunteers were included in the study for the control group (12 females, age: 62.3 ± 9.8). ESUS was defined as a non-lacunar infarct and no apparent embolic etiology (except paroxysmal AF which is detected in Holter monitoring ECG) in patients with stroke. Carotid Doppler ultrasonography and echocardiography showed no carotid stenosis higher than 50% and no cardiac embolic source in all stroke patients. All patients were monitored for more than 24 h with Holter ECG. Then patients were divided into two groups (ESUS + PAF and ESUS-PAF) according to develop of AF in Holter monitoring ECG. ESUS classification was not done with outcomes of 48–72 h of Holter monitoring ECG due to study design and aim. Patients with end-stage kidney disease, active COVID- 19 or another significant infectious disease, advanced systolic heart failure (left ventricle ejection fraction < 35%), and respiratory insufficiency which needs a mechanical respiratory machine or advanced respiratory support were excluded from the study. Stroke severity was assessed by the National Institute of Health stroke scale (NIHSS) and modified Rankin scale (mRS).

### 2.1. Electrocardiography

Participants’ 12-channel surface ECG was obtained after inclusion in this study. The ECG Holter monitoring were performed during hospitalization, 24–96 h after the initial hospitalization. Immediately after echocardiography, patients were monitored with two-channel (five-lead) Holter-ECG-monitoring (GE Healthcare SEERTM 1000, Chalfont St Giles, UK) scheduled for 48–72 h. Paroxysmal AF description based on 2020 ESC Guidelines for the diagnosis and management of atrial fibrillation [13]. The minimum period of at least 30 s in Holter monitoring ECG or complete 12-lead ECG of AF period was accepted as paroxysmal AF.

Leads D2 and V1 were used for measuring of PR interval which was defined as the time to the beginning of the P wave and the beginning of QRS. P wave peak time was accepted as the beginning of the P wave to its peak. P wave time was defined as the time from the beginning to the end of the P wave and measured from leads D2 and V1.

### 2.2. Echocardiography

Echocardiographic examinations of all participants were performed by a cardiology specialist according to recommendations of the American Society of Echocardiography [14]. The left lateral position and apical 2 and 4 cavity images were obtained from the parasternal short and long axes. M-mode was used to measure the left ventricular (LV) end-systolic and end-diastolic diameters from the parasternal long axis (at the mitral chordal level perpendicular to the long axis of the ventricle).

Longitudinal strain analyses of the LA were performed offline (QLab 7.0, Philips Medical Systems, Andover, MA, USA) using zoom mode images of the LA in four and two-chamber views. The point and select method were used to manually trace the endocardial edge of the LA. The region of interest was fitted to the thickness of the atrial myocardium. In each view, the LA was automatically divided into six segments giving longitudinal strain curves from a sum of 12 segments.

Atrial EMD was defined as the time interval from the onset of atrial electrical activity (P wave on surface ECG) to the beginning of mechanical atrial contraction (late diastolic A wave). All values were averaged over 3 consecutive beats. Atrial EMD was measured from the lateral mitral annulus and called ‘PA lateral’, from the septal mitral annulus, called ‘PA septal’, and from the right ventricle tricuspid annulus, called ‘PA tricuspid’ [15].

LAVI was calculated from biplane LA measurements of images obtained in the 4- and 2-chamber views. LAVI measurements using the biplane method of disks (modified Simpson’s rule). LAVI was determined by dividing LA volume by body surface area.

### 2.3. Galectin-3

Venous blood samples were obtained before Holter monitoring ECG and then immediately centrifuged and stored at −80 °C until assayed. The frozen serum samples were quickly melted and carried to room temperature and assayed for the presence of human galectin-3 by using enzyme-linked immunosorbent assay (ELISA) kits (Bioassay Technology Laboratories, Human-Gelctin-3 Elisa Kits, Shanghai, China), according to the manufacturer’s instructions.

### 2.4. Statistical Analyses

The Statistical Package for Social Sciences software program (SPSS, version 25.0 for Windows) was used for statistical analysis. Continuous variables were given as means ± SD; categorical variables were defined as percentages. One-way analysis of variance (ANOVA) and post hoc Tukey test were used to compare the study variables among groups. The χ2 test was used for univariate analysis of the categorical variables. Correlation analyses were performed using Pearson’s coefficient of correlation. A probability value of *p* < 0.05 was considered significant, and 2-tailed *p* values were used for all statistics.

Univariable logistic regression analyses were used for clinical, electrocardiographic, and echocardiographic parameters and serum galectin-3 levels to describe predictors of PAF. The parameters that were found significant in univariable binary logistic regression analyses were included in multivariable logistic regression analyses.

The threshold level of serum galectin-3 which predicts the PAF with optimal sensitivity and specificity, was determined by using the receiver operating characteristics (ROC) curve. The area under the curve (AUC) was calculated from ROC analysis.

## 3. Results

30 patients with ESUS whose Holter ECG monitoring showed PAF, were recruited to the ESUS + PAF group. Other 120 patients with ESUS were recruited to the ESUS-PAF group.

Baseline characteristics are shown in Table 1. Age was similar in groups (*p* = 0.187). All groups’ histories of hypertension, heart failure, and coronary artery disease were not different (*p* = 0.173, *p* = 0.588, and *p* = 0.352, respectively). Diabetes was higher in the ESUS-PAF group and it reached the border significantly (*p* = 0.050). BMI and CHADS-VASc score were not significantly different in groups (*p* = 0.180, *p* = 0.316). Heart rate was similar in groups (*p* = 0.324). The Holter duration was higher in the ESUS + PAF group (47.63 ± 8.85 h vs. 51.27 ± 7.58 h, *p* = 0.041). NIHS score and mRS score were similar in ESUS + PAF and ESUS-PAF groups (5.98 ± 3.45 vs. 6.90 ± 3.05, 1.88 ± 1.07 vs. 2.20 ± 0.925, *p* = 0.186, *p* = 0.139).

Table 2 demonstrates the ECG findings of the groups. PR interval, P wave time, and P wave peak time were higher in the ESUS + PAF group than ESUS-PAF group but the PR interval was not statistically significant (163.2 ± 22.7 ms vs. 170.8 ± 21.3 ms, 98.4 ± 14.3 ms vs. 105.3 ± 17.2 ms, 53.3 ms ± 11.0 ms vs. 59.2 ms ± 12.7 ms, *p* = 0.099, *p* = 0.027 and *p* = 0.013, respectively).

Echocardiographic parameters and serum galectin-3 levels of groups were presented in Table 3. LV systolic and diastolic dimensions were not different in groups (*p* = 0.061 and *p* = 0.090, respectively). Left ventricular interventricular septum and posterior wall thickness were higher in the ESUS + PAF group (*p* = 0.001 for all). LA diameter, LA area, and LAVI were higher in the ESUS + PAF group (*p* < 0.001, for all). Also, atrial electromechanical delay parameters; PA lateral, PA septum, and PA tricuspid were higher in the ESUS + PAF group (*p* < 0.001 for all). Global peak LA strain was lower in the ESUS + PAF group than in other groups (*p* < 0.001).

**Figure 1:**
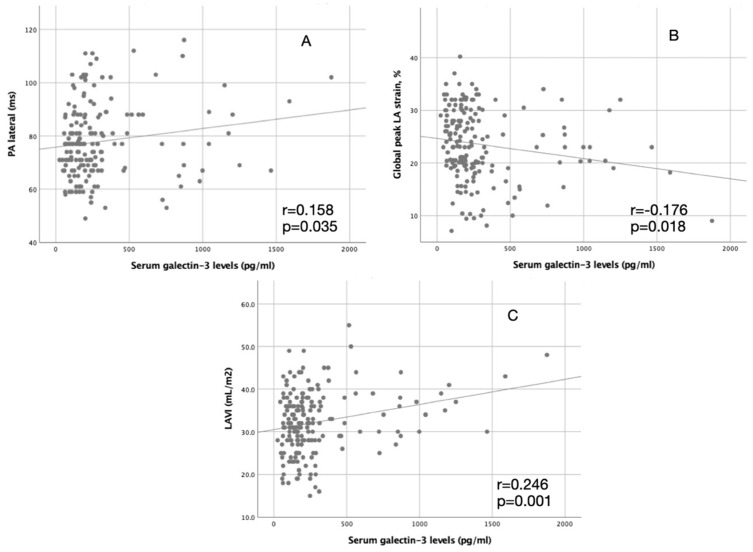
Demonstrates the correlation between serum Galectin-3 levels and PA lateral (**A**), global peak left atrium strain (**B**), left atrial volume index (**C**), LA: left atrium, LAVI: left atrium volume index.

Serum galectin-3 levels were significantly higher in ESUS + PAF than in ESUS-PAF and control groups (479.0 pg/mL ± 435.8 pg/mL, 297.8 pg/mL ± 280.3 pg/mL, and 125.4 ± 87.0 pg/mL, *p* < 0.001, respectively). Serum galectin-3 levels were significantly correlated with LAVI, PA lateral, and global peak LA strain (r = 0.246, *p* = 0.001, *p* = 0.158, *p* = 0.035, r = −0.176, *p* = 0.018, respectively). Table 4 demonstrates correlation analyses of galectin-3 and LAVI, PA lateral, and global peak LA strain. Also, it is shown in a figure (Figure 1).

Univariate and multivariate logistic regression analyses for predictors of PAF are shown in Table 5. In univariate logistic regression analyses, Holter ECG monitoring duration, P wave duration, P wave peak time, global peak LA strain, PA lateral, PA septum, PA tricuspid, LAVI and galectin-3 level were significant predictors of PAF (*p* = 0.043, *p* = 0.029, *p* = 0.015, *p* < 0.001, *p* < 0.001, *p* < 0.001, *p* < 0.001, *p* < 0.001 and *p* = 0.010, respectively). In multivariate logistic regression analyses, global peak LA strain, PA lateral and galectin-3 level can significantly predict PAF (*p* = 0.015, *p* = <0.001 and *p* = 0.048, respectively).

**Figure 2:**
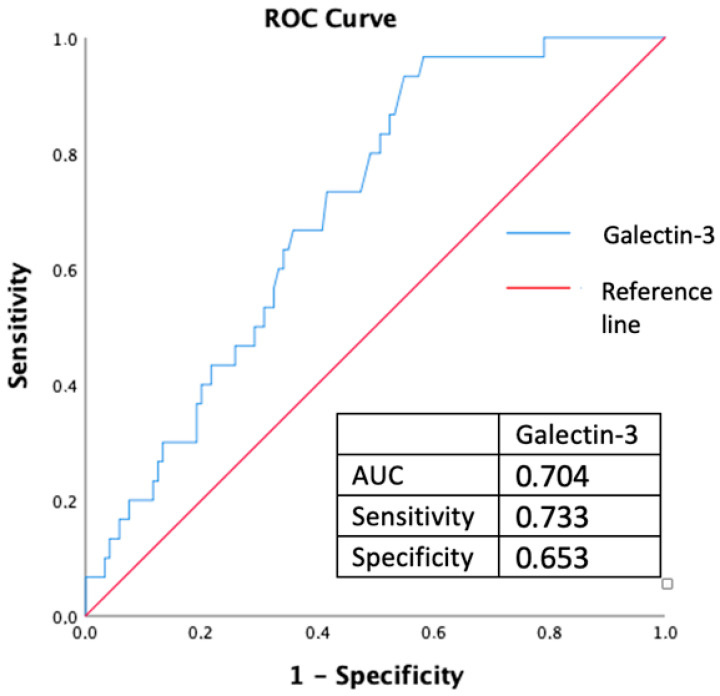
Demonstrating of receiver operating characteristics (ROC) curve of 235.1 pg/mL serum galectin-3 level for predicting paroxysmal atrial fibrillation, AUC: Area under curve.

The optimal threshold of galectin-3 levels for diagnosing PAF was determined by using ROC analysis. 235.1 pg/mL serum galectin-3 levels predict PAF with 0.733 sensitivity and 0.653 specificity (AUC, 0.704; 95%CI, 0.614–0.794; *p* = 0.001) (Figure 2).

## 4. Discussion

In this study, three outcomes that can be important for clinical implications were revealed. First, P wave time and P wave peak time are significantly higher in the ESUS + PAF group. The second result is echocardiographic parameters. Atrial electromechanical delay durations and LAVI independently predict PAF. Another important result is that galectin-3 levels were higher in the ESUS + PAF group and correlated with echocardiographic parameters.

Paroxysmal atrial fibrillation (PAF) is an important cause that is thought to be the main potential factor in ESUS. Randomized controlled trials of prolonged cardiac monitoring like the CRYSTAL-AF (Study of Continuous Cardiac Monitoring to Assess Atrial Fibrillation After Cryptogenic Stroke), EMBRACE (30-Day Cardiac Event Monitor Belt for Recording Atrial Fibrillation After a Cerebral Ischemic Event) showed that AF may be detected in 30% of ESUS patients during long-term follow-up [16,17]. Studies show prolonged monitoring can detect PAF cases that were previously grouped as cryptogenic stroke. Because prolonged monitoring is an inconvenient and expensive tool, every patient cannot receive monitoring with adequate duration. Some ECG and echo parameters are associated with paroxysmal AF.

In ECG, the P wave shows the electrical activity of the sinoatrial node to the atrioventricular node. Larger atrium or electrical delays deteriorate P wave morphology. Yildirim et al. showed that PR interval, P wave duration, and P wave peak time are longer in paroxysmal AF [18]. Another study showed that P wave peak time is associated with paroxysmal AF in patients with stroke [12]. However, P wave morphologic changes have not been studied in patients with ESUS yet. In our study, P wave duration and P wave peak time were longer in the ESUS + PAF group. Prolongation of P wave duration and P wave peak time in patients with ESUS can be associated with PAF [19]. P wave peak time and P wave duration have prognostic value in coronary artery disease, heart failure and PAF. Prolonged P wave peak time and duration may associate recurrence of stroke recurrence in patients with ESUS.

In our study, LAVI is an independent predictor for developing PAF in Holter ECG. LA enlargement is thought to be a risk factor for occurring AF and thrombus formation due to electrical instability, stasis, and endothelial dysfunction [20]. Jordan K et al. showed that LAVI is an independent risk factor for cardioembolic stroke and PAF [9]. Our finding supports Jordan et al.’s study results [9]. Because LAVI was shown to be associated with diastolic dysfunction in hypertensive patients, LAVI can increase in patients with PAF [21]. LAVI measurement can be a utility marker in clinical practice for determining the risk status of PAF.

Atrial electromechanical delay, which is an easy and reproducible method, has been studied previously in many clinical situations as a marker for developing PAF [15,22]. In a study, septal atrial electromechanical delay was found superior to other echocardiographic parameters for predicting PAF in patients with ESUS [10]. We found that atrial electromechanical delay was higher in the ESUS-PAF group. Also, PA lateral, PA septal, and PA tricuspid are associated with developing AF. These findings support atrial EMD measurements are useful in patients with ESUS for predicting new onset of AF.

Galectin-3 is a protein that has a role in adhesion and proliferation of cells and immunity [23]. It was associated with worsening heart failure, coronary artery disease, and hypertrophic cardiomyopathy [24,25]. Galectin-3 was associated with LA remodeling which occurred in AF [8]. Yalcin et al. found that galectin-3 was correlated with atrial EMD parameters and the extent of LA fibrosis [8]. In the ARIC study, galectin-3 levels were significantly higher in patients with AF, but the significance decreased in patients with AF along with heart failure and coronary artery disease [26]. Clementi N showed that galectin-3 levels associated with LA diameter and higher galectin-3 levels predict AF recurrence in patients who performed AF ablation [27]. In our study, heart failure and coronary artery disease frequency were similar in ESUS-PAF and ESUS + PAF groups, the elevated galectin-3 levels in ESUS + PAF group may be due to adverse LA remodeling and LA fibrosis. Also in univariate regression analyses, galectin-3 levels predict PAF more strongly than multivariate regression analyses (*p* = 0.010 vs. *p* = 0.048). This is probably due to correlation between galectin-3 and atrial echo remodeling markers (LAVI, LA strain and atrial conduction delay).

A higher concentration of galectin-3 is associated with higher stroke severity and poor prognosis [26].It was thought to result from the upregulation response of galectin-3 to hypoxia-ischemic of the brain [28]. In our study, mRS and NIHSS were similar in the ESUS + PAF and ESUS-PAF groups. LA diameter and LAVI were higher in the ESUS + PAF group. Considering the significant correlation between galectin-3 levels and PA lateral, LAVI, and global peak LA strain, serum galectin-3 levels should originate from the heart. These results thought that elevated galectin-3 levels in ESUS patients are riskier for PAF.

Some limitations should be considered. 48–72 h Holter ECG monitoring was used for AF detection. Some patients with PAF might be not detected in 48–72-h ECG monitoring [13]. Implantable cardiac monitors or some smartwatches can screen longer duration for AF. If we used these kinds of tools for screening, we could detect more patients with PAF. Other limitation is a relatively small sample size. If we performed the study in a higher number of patients, the results could be interpreted more accurately, and galectin-3 levels might have predicted PAF better. Another limitation is Holter ECG monitoring time were slightly higher in ESUS + PAF group more than ESUS-PAF group. Longer duration of Holter ECG-monitoring could have detected more PAF patients.

## 5. Conclusions

In this study, P wave duration, P wave peak time, LAVI, PA lateral, global LA strain are higher in patients with ESUS + PAF compared to patients with ESUS-PAF and the control group. Serum galectin-3 levels correlated with PA lateral, LAVI, and global peak LA strain. Serum galectin-3 levels are associated LA adverse remodeling in patients with ESUS.

## Figures and Tables

**Table 1:** Baseline characteristics of groups.

	ESUS-PAF Group	ESUS + PAF Group	Control Group	*p* Value *	*p* Value **
Age	64.16 ± 11.88	67.53 ± 9.60	62.37 ± 9.82	0.152	0.187
Female sex, (n%)	56 (46%)	13 (43%)	12 (40%)	0.839	0.790
Diabetes, (n%)	45 (37.5%)	17 (56%)	8 (26%)	0.065	0.050
Heart Failure, (n%)	9 (7.5%)	3 (10%)	4 (13.3%)	0.707	0.588
Coronary artery disease, (n%)	29 (24%)	11 (36%)	7 (23%)	0.173	0.352
Hypertension (n%)	84 (70%)	26 (86%)	21 (70%)	0.070	0.173
BMI (kg/m^2^)	25.35 ± 3.25	26.31 ± 4.24	25.79 ± 3.42	0.180	0.380
CHADS-VASc Score	3.06 ± 1.71	3.40 ± 1.45	2.27 ± 1.63	0.316	
Heart Rate (beats/min)	73.41 ± 12.35	75.90 ± 12.21	74.43 ± 12.37	0.324	0.600
Holter duration (h)	47.63 ± 8.85	51.27 ± 7.58	NA	0.041	NA
NIHSS	5.98 ± 3.45	6.90 ± 3.05	NA	0.186	NA
mRS	1.88 ± 1.07	2.20 ± 0.925	NA	0.139	NA

NIHS: National Institutes of Health stroke scale; mRS: modified Rankin scale; * *p* value between ESUS-PAF and ESUS + PAF groups, ** *p*-value between all groups.

**Table 2:** ECG findings of groups.

	ESUS-PAF Group	ESUS + PAF Group	Control Group	*p* Value *	*p* Value **
PR interval (ms)	163.2 ± 22.7	170.8 ± 21.3	155.2 ± 16.8	0.099	0.021
P wave time (ms)	98.4 ± 14.3	105.3 ± 17.2	89.9 ±16.7	0.027	0.001
P wave peak time (ms)	53.3 ± 11.0	59.2 ± 12.7	49.3 ± 8.7	0.013	0.002

* *p* value between ESUS-PAF and ESUS + PAF groups, ** *p*-value between all groups.

**Table 3:** Echocardiographic parameters and serum galectin-3 levels of groups.

	ESUS-PAF Group	ESUS + PAF Group	Control Group	*p* Value *	*p* Value **
LVSd (cm)	3.36 ± 0.49	3.58 ± 0.84	3.33 ± 0.31	0.061	0.106
LVDd (cm)	4.89 ± 0.59	4.67 ± 0.83	5.05 ± 0.44	0.090	0.061
IVSd (cm)	1.06 ± 0.16	1.18 ± 0.14	1.05 ± 0.13	<0.001	<0.001
PWd (cm)	1.01 ± 0.14	1.12 ± 0.13	1.01 ± 0.14	<0.001	0.001
LVEF (%)	62.9 ± 3.65	64.0 ± 4.14	62.73 ± 3.64	0.153	0.296
LA diameter (cm)	3.41 ± 0.36	3.80 ± 0.36	3.30 ± 0.31	<0.001	<0.001
LA area (cm^2^)	13.56 ± 3.26	18.38 ± 4.09	12.36 ± 2.54	<0.001	<0.001
LA Volume Index (mL/m^2^)	32.13 ± 6.84	39.06 ± 5.93	26.00 ± 4.57	<0.001	<0.001
PA lateral (ms)	73.94 ± 10.74	97.83 ± 10.95	73.7 ± 7.56	<0.001	<0.001
PA septum (ms)	64.01 ± 10.75	84.80 ± 11.23	61.83 ± 5.94	<0.001	<0.001
PA tricuspid (ms)	54.76 ± 10.19	75.27 ± 11.02	51.33 ± 5.96	<0.001	<0.001
LA global peak strain (%)	23.68 ± 6.30	16.87 ± 4.51	29.40 ± 3.61	<0.001	<0.001
Serum galectin-3 (pg/mL)	297.8 ± 280.3	479.0 ± 435.8	125.4 ± 87.0	0.006	<0.001

LVSd: left ventricle systolic diameter; LVDd: left ventricle diastolic diameter; IVSd: interventricular septum diameter; PWD: posterior wall diameter; LVEF: left ventricular ejection fraction; LA: left atrium; * *p* value between ESUS-PAF and ESUS + PAF groups, ** *p*-value between all groups.

**Table 4:** Correlation analysis between serum galectin-3 levels and left atrial volume index, PA lateral.

	LAVI (mL/m^2^)		PA Lateral (ms)		Global LA Strain%	
	r	*p*	r	*p*	r	*p*
Serum galectin-3 levels (pg/mL)	0.246	0.001	0.158	0.035	−0.176	0.018

LAVI: left atrium volume index, LA: left atrium.

**Table 5:** Predictors for AF in ESUS patients.

	Univariate Regression Model			Multivariate Regression Model		
	OR	95% CI	*p* Value	OR	95% CI	*p* Value
Age (year)	1.028	0.990–1.068	0.153			
Female Sex	0.874	0.390–1.957	0.743			
Heart failure	1.370	0.347–5.407	0.653			
Hypertension	2.786	0.906–8.561	0.074			
Diabetes	2.179	0.968–4.905	0.060			
Coronary artery disease	1.817	0.775–4.259	0.170			
NIHSS	1.080	0.963–1.210	0.187			
mRS	1.316	0.913–1.898	0.141			
CHADS-VASc Score	1.130	0.891–1.433	0.315			
PR interval (ms)	1.015	0.997–1.033	0.102			
Holter Monitoring Duration (h)	1.050	1.002–1.101	0.043	1.080	0.978–1.192	0.127
P wave duration (ms)	1.032	1.003–1.061	0.029	0.962	0.891–1.039	0.323
P wave peak duration (ms)	1.045	1.009–1.083	0.015	0.962	0.891–1.039	0.203
Global Peak LA Strain%	0.821	0.754–0.894	<0.001	0.821	0.700–0.963	0.015
PA Lateral (ms)	1.206	1.130–1.287	<0.001	1.500	1.194–1.885	<0001
PA Septal (ms)	1.190	1.118–1.267	<0.001	0.739	0.545–1.002	0.052
PA Tricuspit (ms)	1.196	1.123–1.273	<0.001	1.130	0.906–1.409	0.279
LAVI (mL/m^2^)	1.180	1.094–1.273	<0.001	0.920	0.798–1.060	0.248
Serum galectin-3 levels (pg/mL)	1.001	1.000–1.003	0.010	1.003	1.000–1.006	0.048

NIHSS: National Institutes of Health stroke scale; mRS: modified Rankin scale, LA: left atrium, LAVI: left atrium volume index.

## Data Availability

Data are contained within the article.
